# Nano/Micro-Assisted Regenerative Medicine

**DOI:** 10.3390/ijms19082187

**Published:** 2018-07-26

**Authors:** Bogyu Choi, Soo-Hong Lee

**Affiliations:** Department of Biomedical Science, CHA University, 335 Pangyo-ro, Bundang-gu, Seongnam-si 13488, Korea; bgchoi@cha.ac.kr

Regenerative medicine is an emerging discipline aimed at repairing and reestablishing the normal functions of tissues and organs damaged by aging, disease, injury, or congenital disorders. Among the advanced technologies currently under investigation, such as cell therapy, tissue and biomaterial engineering, transplantation, nano/microtechnologies, either alone or in combination with specific cells, such as stem cells, have opened the prospect of nano/micro-assisted regenerative medicine, which has the potential to transform regenerative medicine.

This special issue, entitled “Nano/Micro-Assisted Regenerative Medicine” presents two reviews and 11 research articles highlighting recent advances in the use of nano/micro-assisted technologies in regenerative medicine. Kang et al. describe the application of nano and microengineering techniques for the fabrication of native tissue topographies as an alternative to silicone implants, which are known to cause capsular contractures via adverse immune reactions [1]. Bellinghen et al. report that temporomandibular joint regeneration can be improved by nano/micro-assisted functionalization [2]. Yun et al. show that labeling mesenchymal stem cells (MSCs) with superparamagnetic iron oxide nanoparticles (SPIONs) via magnetic retention enhances the homing efficiency of MSCs in olfactory-injured mice [3]. Jeong et al. describe the therapeutic effects of simvastatin-loaded porous microspheres (SIM/PMSs) on inflamed tenocytes in vitro and collagenase-induced Achilles tendinitis in vivo [4]. A new platform of three-dimensional (3D) graphene/arginine-glycine-aspartic acid (RGD) peptide nanoisland composites to enhance the osteogenesis of human adipose-derived MSCs is proposed by Kang et al. [5]. Lee et al. show that acid-degradable poly(ethylene glycol)-poly(amino ketal) (PEG-PAK)-based micelles can be used to improve stromal cell-derived factor-1α (*SDF-1α*) gene transfection efficacy and angiogenesis of human adipose-derived MSCs for the treatment of ischemic diseases [6]. Kim et al. demonstrate that a reduced graphene oxide-coated biphasic calcium phosphate bone graft material is effective for bone regeneration in rat calvarial defects [7]. Müller et al. demonstrate that amorphous polyphosphate nano/microparticles effectively block the neurotoxic effects of toxic amyloid β-protein fragment 25–35 by rebalancing the β-amyloid-induced decrease in adenosine triphosphate (ATP) levels [8]. Park et al. describe the development of in vitro cancer microtissue arrays on a fibroblast-layered nanofibrous membrane by inkjet printing and their applications to cancer drug screening and gradual 3D cancer studies [9]. Nagai et al. demonstrate that cilostazol ophthalmic nanodispersions have therapeutic effects on retinal disorders caused by diabetes mellitus in streptozotocin-induced diabetic rats [10]. Tatiparti et al. report the development of the carbonic anhydrase-IX selective nanocarrier, human serum albumin-paclitaxel-acetazolamide (HSA-PTX-ATZ), by copper-free ‘click’ chemistry-based synthesis for tumor hypoxia-targeted drug delivery that can be adapted to several types of cancers [11]. The cytotoxicity of peptide liposome incorporated citron-extract nanoparticles and turmeric extract incorporated oil-in-water nanoemulsions on various cell types is evaluated by Zhang et al. [12] and Yoon et al. [13], respectively.

Regenerative medicine is constantly evolving from advances in the development of new nano/micro-based materials, such as particles, fibers, composites, and surfaces. This evolution is bolstered by the multidisciplinary and interdisciplinary efforts of scientists in areas such as biotechnology, biomaterials science, chemistry, physics, stem cell biology, developmental biology, and clinical medicine, as well as other areas. In this special issue, promising applications of nano/micro-assisted regenerative medicine in tissue engineering or cancer treatment are introduced, and strategies for the further development of this field are described. We are confident that progress in nano/microtechnologies will continue to fertilize the emerging field of nano/micro-assisted regenerative medicine and provide a wide range of new and improved therapies for the degenerative disease.
